# Family Physician Perceptions of Climate Change, Migration, Health, and Healthcare in Sub-Saharan Africa: An Exploratory Study

**DOI:** 10.3390/ijerph18126323

**Published:** 2021-06-11

**Authors:** Charlotte Scheerens, Els Bekaert, Sunanda Ray, Akye Essuman, Bob Mash, Peter Decat, An De Sutter, Patrick Van Damme, Wouter Vanhove, Samuel Lietaer, Jan De Maeseneer, Farai Madzimbamuto, Ilse Ruyssen

**Affiliations:** 1 Department of Public Health and Primary Care, Ghent University, B-9000 Ghent, Belgium; Peter.Decat@UGent.be (P.D.); An.DeSutter@UGent.be (A.D.S.); Jan.DeMaeseneer@UGent.be (J.D.M.); 2 Department of Economics—CESSMIR, Ghent University, UNU-CRIS, B-9000 Ghent, Belgium; Els.Bekaert@UGent.be (E.B.); Ilse.Ruyssen@UGent.be (I.R.); 3 Department of Lung Health, BIDMC, Harvard Medical School, Boston, MA 02215, USA; 4 Department of Medical Education, Faculty of Medicine, University of Botswana, Gaborone, Botswana; sunanda28@hotmail.com; 5 School of Public Health, University of Witwatersrand, Johannesburg, South Africa; 6 Department of Community Health, University of Ghana, Accra, Ghana; AEssuman@ug.edu.gh; 7 Division of Family Medicine and Primary Care, Faculty of Medicine and Health Sciences, Stellenbosch University, Cape Town 7505, South Africa; rm@sun.ac.za; 8 Department of Plants and Crops, Ghent University, B-9000 Ghent, Belgium; Patrick.VanDamme@UGent.be (P.V.D.); Wouter.Vanhove@UGent.be (W.V.); 9 Department of Environmental management and Land-use Planning, IGEAT-Centre d’Etudes du Développement Durable (CEDD), Université Libre de Bruxelles, B-1050 Brussels, Belgium; samlietaer@hotmail.com; 10 Department of Anaesthetics and Critical Care, University of Botswana, Gaborone, Botswana; faraitose@hotmail.com

**Keywords:** climate change, migration, family doctors, Sub-Saharan Africa, primary care, healthcare, health

## Abstract

Although family physicians (FPs) are community-oriented primary care generalists and should be the entry point for the population’s interaction with the health system, they are underrepresented in research on the climate change, migration, and health(care) nexus (hereafter referred to as the nexus). Similarly, FPs can provide valuable insights into building capacity through integrating health-determining sectors for climate-resilient and migration-inclusive health systems, especially in Sub-Saharan Africa (SSA). Here, we explore FPs’ perceptions on the nexus in SSA and on intersectoral capacity building. Three focus groups conducted during the 2019 WONCA-Africa conference in Uganda were transcribed verbatim and analyzed using an inductive thematic approach. Participants’ perceived interactions related to (1) migration and climate change, (2) migration for better health and healthcare, (3) health impacts of climate change and the role of healthcare, and (4) health impacts of migration and the role of healthcare were studied. We coined these complex and reinforcing interactions as continuous feedback loops intertwined with socio-economic, institutional, and demographic context. Participants identified five intersectoral capacity-building opportunities on micro, meso, macro, and supra (international) levels: multi-dimensional and multi-layered governance structures; improving FP training and primary healthcare working conditions; health advocacy in primary healthcare; collaboration between the health sector and civil society; and more responsibilities for high-income countries. This exploratory study presents a unique and novel perspective on the nexus in SSA which contributes to interdisciplinary research agendas and FP policy responses on national, regional, and global levels.

## 1. Introduction

The climate change, migration, and health nexus has been extensively explored in and beyond the Sub-Saharan Africa (SSA) region [1,2,3,4]. Explanatory frameworks highlighting the linkages together with the feedforward and feedback relationships are useful to inform and guide the formulation of research agendas and policy responses [1,2,3,5].

Although SSA has the lowest share of greenhouse gases compared to that of high-income countries, it is most vulnerable to impacts of anthropogenic climate change [5]. This is partly due to its strong reliance on rainfed agriculture and fisheries/aquaculture [6,7]. Climate disasters in SSA may further affect and exacerbate socio-economic inequities, food insecurity, shelter, access to safe water and sanitation, and increased conflict over scarce resources [1,8,9]. They may also limit access to public health services [10], with potentially increasing disastrous health hazards [1,8,9,10]. This may lead to changing migration patterns, with further health implications and reduced healthcare access [9,11]. Until now, these implications have remained “underexplored and undertheorized” [3] (p. 217).

Public sector health facilities in SSA suffer from understaffing, poor infrastructure, geographical maldistribution, and an inappropriate skill mix [12]. Austerity measures imposed by some international donors, and reduced public health expenditure in many SSA countries, have led to underfinancing, under-resourcing, and donor dependency, compromising the health sectors’ ability to provide effective and equitable healthcare [13,14]. A shift to strengthened, climate-resilient, migration-inclusive health systems may better anticipate, prevent, prepare for, monitor, and manage changing health needs [4,9]. To achieve this, international bodies suggest an “intersectoral capacity-building approach”. This means integrating capacity in health-related sectors such as healthcare itself, disaster management, urban planning, education, water and sanitation, and food production [15,16].

Family physicians (FPs) in SSA are often based in district hospitals and primary care facilities [17]. These facilities may be in the public, faith-based, or private for-profit sectors. As community-oriented primary healthcare (PHC) generalists, they are well placed to oversee health teams engaging with local communities to improve health [17]. PHC is the entry point for the population’s interaction with the health system. It is crucial for strengthening national and community responses to climate change and migration [18,19]. Still, PHC providers are often absent in the debate on how to support and prevent health outcomes related to climate change and migration [20]. Exploring the knowledge, awareness, and insights of PHC providers on the nexus and health system strengthening is needed for public health interventions and climate-resilient and migrant-inclusive health systems, to include their leadership and advocacy [18]. We hypothesize that FPs will reveal a unique and novel set of perceived interactions in and perspectives on the nexus in SSA and on intersectoral capacity-building opportunities.

## 2. Materials and Methods

### 2.1. Study Design

The study used a descriptive exploratory qualitative design with focus group discussions (FGDs), conducted during the World Organisation of Family Doctors (WONCA) 2019 regional conference in Kampala, Uganda. FGDs were chosen because their group dynamic stimulates interaction and exploration of the topic. Ghent University Hospital provided ethics approval (reference B670201940365).

### 2.2. Setting

The WONCA-Africa 2019 regional conference in Kampala brought together FPs, health professionals, and academics from SSA working in PHC. The Primary Care and Family Medicine Network for SSA (https://primafamed.sun.ac.za/, accessed on 10 June 2021) held a pre-conference meeting during which the study was conducted.

### 2.3. Selection of Participants

Registrants for the Primafamed pre-conference and WONCA conference were invited by e-mail and then in person during the pre-conference to join the FGDs if they met the inclusion criteria. These were: (1) residence in SSA, (2) involvement in PHC, and (3) being English or French speaking. Of all the attendees at these two conferences, 30 self-selected to join the FGDs. Participants gave written informed consent, completed a short demographic questionnaire, and were divided into three FGDs.

### 2.4. Data Collection

A semi-structured topic guide was developed (Box 1), with topics selected from relevant literature [1,2,3,9] and aligned with the study objectives. The study was introduced by IR, while EB and PD took notes, and CS and JDM facilitated. The FGDs took place non-concurrently in a quiet room, lasted 100–120 min, and were audio recorded. One FGD was conducted with only English speakers; the other two with English and French speakers. The moderator (JDM) translated questions into French and translated answers given in French to English for the other participants.

Box 1 **Key topics in the interview guide.**

**Part I: Health(care) and climate change as drivers of migration**

1/ Health(care) as a driver for migration

2/ Climate change as a driver for migration

3/ The indirect impact of climate change on migration through health(care)

4/ What kind of capacity-building would you indicate as most helpful?

**Part II: The health(care) impacts of climate change and climate-related migration**

5/ Health(care) impact of climate change

6/ Health(care) impact of migration

7/ Healthcare impact of climate-related migration, displacement, and relocation (on host and home community)

8/ What kind of capacity-building would you indicate as most helpful?

### 2.5. Data Analysis

An inductive thematic approach was used for data analysis. The FGDs were transcribed verbatim. French was translated into English and doublechecked by EB. The Ghent University team initially read through the transcripts to familiarize themselves with the data. They then read through the first transcript independently and identified critical themes of interest. They discussed and agreed upon a coding framework that was used to independently analyze all transcripts. The group added new codes during this process by consensus. Codes were then clustered into groups around similar topics and interpreted for key themes emerging from the coded data. For reflections on trustworthiness, see Box 2.

Box 2 **Trustworthiness of the data analysis.**

To ensure trustworthiness of the analysis, two authors from the University of Botswana (SR and FM), who were not part of the FGDs or the Ghent University team, conducted separate and independent manual coding of the transcripts for comparison with the primary coding framework. The variations from the original coding and thematic analysis were resolved through further discussion with the full research team to reach consensus. There were several virtual follow-up meetings with all the authors to discuss the analysis and interpretation of the findings. To ensure methodological rigor and creditability of the results and of the study, all the authors were transparent about their own views and opinions and how these related to the interpretation of the findings. The team included researchers of multidisciplinary backgrounds (economics, health sciences, primary care, and bioengineering) who brought diverse perspectives to the data and during the follow-up discussions. In relation to the FGDs, JDM, as a founder member of Primafamed, was well known to the participants while the other facilitators were not known. In relation to the research topic, JDM and PD have expertise in PHC, CS has expertise in climate change and health, while EB and IR have expertise in climate change and migration. SR and FM have expertise in southern African health systems and in health profession education. SR is part of the Primafamed network and has expertise in PHC research and practice.

## 3. Results

### 3.1. Participant Characteristics

**Figure 1:**
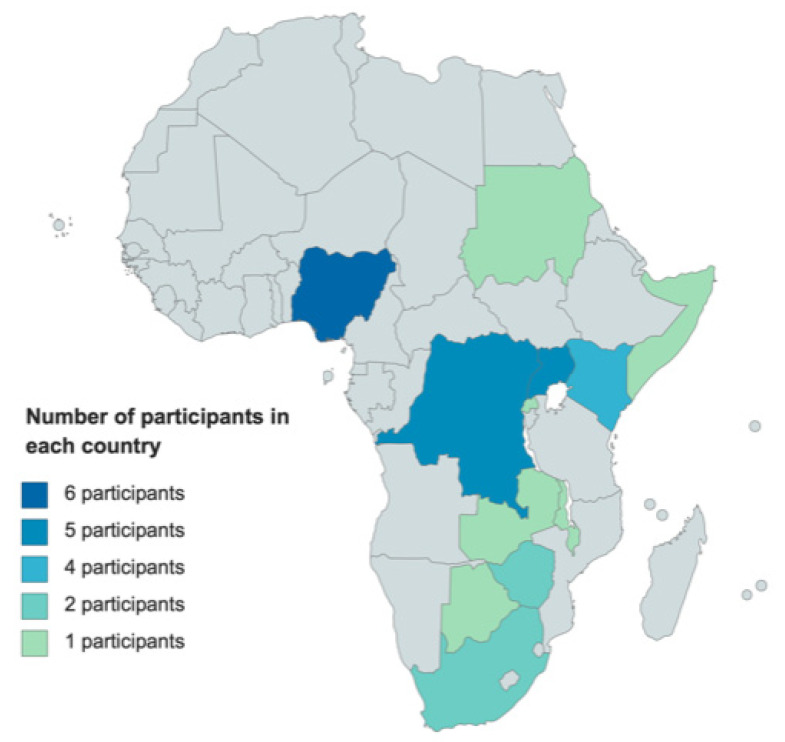
Geographical distribution of participants’ country of residence. Number of participants in each country: Nigeria (6), Democratic Republic of Congo (5), Uganda (5), Kenya (4), Zimbabwe (2), South Africa (2), Sudan (1), Rwanda (1), Zambia (1), Botswana (1), Malawi (1), Somaliland (1).

We interviewed 30 participants from twelve countries in SSA (Figure 1). Twenty-three of them were family physicians and four were doctors training to become specialists in family medicine. The remaining three were a medical student and two non-medical persons involved in PHC. Table 1 provides the demographic profile of the participants and Figure 1 the geographical spread. The findings hereunder are presented as a series of themes, with supportive quotations provided in Table 2.

### 3.2. Perceptions of Migration Related to Climate Change

Participants perceived that slow onset hazards (changed rain and seasonal patterns, droughts, desertification) are impacting subsistence farmers around waterbodies that are drying up. This lowers crop productivity, leads to crop failures and eventually malnutrition and famine. Inability to grow crops for food and income invokes forced (seasonal/temporal) migration for income to survive (quote A). Pastoral communities deviate from their nomadic routes towards game parks in search of better grazing lands for their farm animals. This results in anthrax and foot-and-mouth disease transmission and zoonosis risks. It may also induce interpersonal violence; for example, Nigerian cattle herders occupied fertile farmland and forced farmers to move to cities for their safety (quote B).

Sudden onset hazards such as cyclones and flooding often destroy entire areas. The 2019 Cyclone Idai that hit Beira (Mozambique) exemplifies this. People lost their possessions, homes, and income, and were unable to rebuild their lives, spurring migration. Underlying reasons for moving included a lack of safety nets due to gray economy practices, combined with poor water sanitation systems that were unable to solve contaminated water issues (quote C).

Participants also mentioned soil degradation due to poor agricultural practices. Deforestation for firewood collection and commercial timber production causes landslides after excessive rainfall which, combined with monocultures, degrades the ecosystem through soil erosion and land degradation. All these factors reduce crop productivity and worsen poverty and food insecurity, leading to migration for better access to food and water for household consumption and agricultural use (quote D).

### 3.3. Perceptions of Migration Related to Seeking Better Health and/or Healthcare

Participants describe temporary relocation due to unavailable or unaffordable healthcare treatments in their own countries (for example, surgery conducted in India). Participant 8 mentioned that Nigerian borders are porous with few constraints for refugees to cross borders in search of better healthcare and food security. When sudden (climatic) events with a certain magnitude occur, such migration may overwhelm refugee centers and local health services. Comprehensive health and social service programs (externally funded or not) including medical treatment, food, shelter, and school fees attract people but often lack the resources to cover increased demands. Strikingly, migrants may purposely infect themselves with HIV to be granted access to comprehensive HIV programs that provide childcare services and healthcare for HIV-positive persons (quote E.1.). Similarly, people cross borders for access to enhanced quality healthcare in Uganda; for example, Sudanese women with obstetric complications seeking better maternal and reproductive health. Sometimes people are reluctant to return to their country of origin once they have experienced improved healthcare facilities abroad. Relocation to settlements near health facilities also results in concentrated populations close to hospitals that may put an unsustainable burden on the habitat and resources (quote E.2.).

Importantly, some participants state that local healers continue to perform their traditional healthcare practices. They are typically more accessible, especially in rural areas where hospitals are often scarce. People often only turn to allopathic medicine when traditional remedies have failed (quote F).

### 3.4. Perceptions of Health Impacts Related to Climate Change and the Role of Healthcare

Participants mentioned that droughts and changing rain patterns threaten water security which reduces high-quality food crop production. This induces malnutrition, particularly among children (who risk becoming stunted) and the elderly. Similarly, they attribute the rising prevalence of malaria and meningitis to increasing temperatures.

Health facilities in SSA often lack adequate resources to cope with outbreaks of infectious diseases. Climate disasters can damage health facilities and result in delayed medical support for affected individuals. Patients also come to health facilities at more advanced disease stages (quote G). Families with limited access to health services whose livelihoods are disrupted become further impoverished through out-of-pocket expenses, especially for maternal and child health.

Excessive rainfall, floods, and cyclones, often following droughts, flood pit latrines and contaminate shallow wells. This results in water-borne diseases such as typhoid and cholera. Water stagnation in hot conditions, such as in irrigated rice paddies, enhances mosquito breeding and malaria transmission. Water shortages require rationing and reduce hygienic standards, resulting in diarrheal diseases. Continuous overflooding of riverbanks results in waterlogged areas and the river water increases the prevalence of parasite-infested freshwater snails. Children playing in these waterlogged areas get infected with schistosomiasis, which had previously been eliminated to the extent that younger clinicians are not acquainted with the disease. This can delay accurate diagnosis and adequate treatment (quote H).

Infrastructural repairs to health services, which may be damaged by climate events such as floods, take time and often rely on external donor funding. This may create difficulties in accessing medication for people with chronic conditions such as HIV or hypertension. Stress, anxiety, and depression after floods and cyclones were observed when relatives’ bodies could not be retrieved and when communities vanished completely (quote I).

Health workers functioning in affected remote areas often experience stress, and feel unsupported and isolated. Rural recruitment and retention of health workers is difficult, transport is challenging, basic equipment and infrastructure are often missing, and poor education opportunities complicate raising children. Brain drain may occur when climate disasters cause further disruption, leading to health center closures, exacerbating the healthcare deficit, and undermining community recovery after a disaster (quote J).

### 3.5. Perceptions of Health Impacts Related to Migration and the Role of Healthcare

Rural–urban migration, whether through gradual relocation or precipitated by catastrophes, pressurizes urban health services and infrastructure, as well as safe water supply and sanitation. Health services in the public sector often lack resources to respond to this increased demand. An example is (seasonal) movements of cattle-herders from grazing areas or cattle posts to villages (quote K).

Urban migration or forced displacement into camps leads to overcrowding, poor sanitation, water shortages, over-demanded water infrastructure, and contamination from open sewers to shallow wells, which may cause typhoid and cholera. Rural–urban relocation may expose children to air pollution, which induces respiratory infections and asthma (quote L).

Urban migration not resulting in the anticipated economic opportunities that were often the motivation for moving there has been related to homelessness and eventually substance abuse, and children dropping out of school and risking sexual exploitation. Adolescents from displaced and broken families may look for money to buy food, whereby transactional sex increases the risk of sexually transmitted conditions including HIV, unintended pregnancies, and gender-based violence. Xenophobic attacks present a risk to migrants’ personal security and (mental) health. This may cause them to return home despite the economic disadvantages this could present (quote M).

Relatives may provide resources for migrants or allow migrating families in their city homes. This reliance often induces family crises and desperation, and sometimes depression, increased alcohol abuse, drug abuse, or suicide (quote N).

International and rural–urban health worker brain drain for better clinical facilities, economic opportunities, and education for children or postgraduate training continuously threatens health services. A large influx of migrants can then leave the remaining workforce unable to cope, resulting in frequent sick leave. This reinforces brain drain and healthcare-related migration which puts communities at risk (quote O).

### 3.6. Complex, Interacting, and Continuous Feedback Loops

Participants perceived the nexus’s interactions as continuous and reinforcing feedback loops, leading to accumulating health risks, migration, and disrupted health services. In Zimbabwe, the 2019 Cyclone Idai impacted health and further weakened the healthcare system, leading to outward migration. This weakened the host countries’ health systems, impacting host communities’ and migrants’ health (quote P).

**Figure 2:**
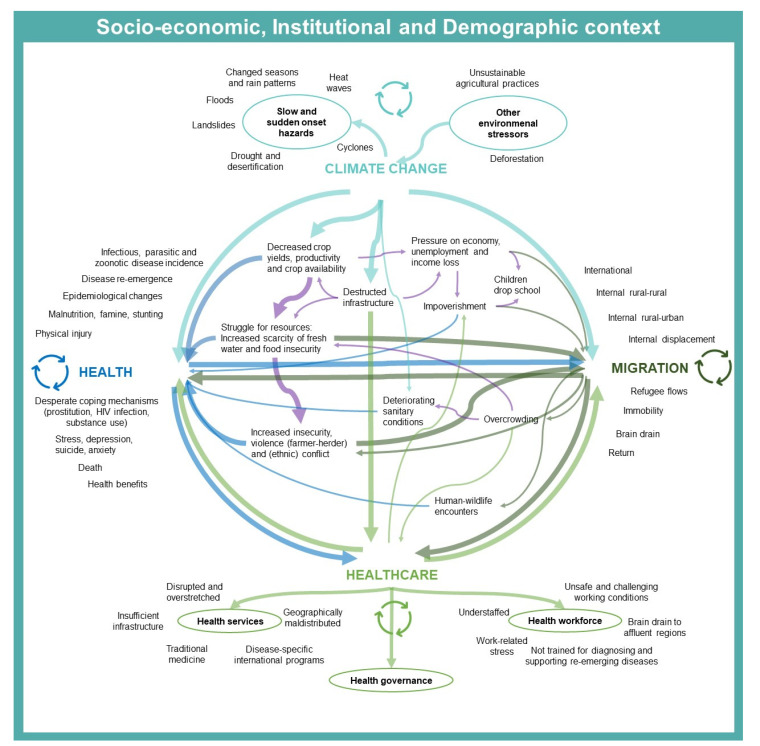
This diagram presents the authors’ interpretation of the participants’ perceptions regarding the climate change, migration, and health(care) nexus in Sub-Saharan Africa. The arrows in the center of the figure denote the direct and indirect interactions between the dimensions identified by the participants. These interactions reinforce each other, resulting in continuous feedback loops between both the nexus’s dimensions and the context-related determinants of health in the middle of the figure. Each arrow denotes a simple perceived impact and/or feedback loop interaction between two components corresponding to at least one participant statement. A sequence of arrows represents indirect interactions (hence a combination of direct interactions) running between interconnected components. The arrow’s thickness is proportionate to the number of quotes on the interactions, corresponding to at least one participant’s statement. The color represents the four dimensions of the nexus, with purple denoting other factors influencing the nexus. The loops denote feedback loops.

The extent and nature of feedback loops may depend on demographic, socio-economic, and political contexts. Poorer regions with more vulnerable healthcare facilities may experience worse climate-related health impacts. In countries with high unemployment, migrant farmers may have less work opportunities in cities, which increases risks of impoverishment. Furthermore, land degradation, overcrowding, deteriorating sanitation, and climate-related water and food insecurity are socio-economic and ecological determinants of health which interact with the feedback loops (quote C) visualized in Figure 2.

### 3.7. Perceived Opportunities for Intersectoral Capacity Building

**Figure 3:**
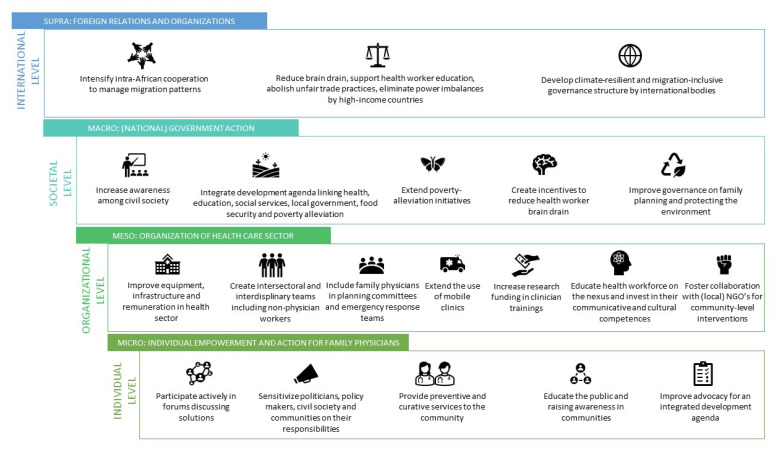
Visualizing intersectoral capacity-building opportunities in the Sub-Saharan African context, on micro, meso, macro, and supra levels.

Participants were asked about intersectoral capacity-building opportunities that could create more climate-resilient and migrant-inclusive health systems, and in particular the role of PHC. Figure 3 visualizes a summary of perceived opportunities at individual and family (micro), community/organizational (meso), societal (macro), and international levels (supra).

#### 3.7.1. Developing Multi-Dimensional and Multi-Layered Governance Structures

Participants urgently called for multi-dimensional and multi-layered governance structures. An integrated development agenda designed by and in collaboration with international bodies and local and national governments can enable intersectoral teamwork between professionals active in PHC, public health, agriculture, water and sanitation, education, social services, local government, food security, and nutrition. This could strengthen policies on nutrition rehabilitation in refugee camps, incentives to reduce brain drain, extend the use of mobile clinics, and inclusion of FPs in country health committees and emergency response teams (quote Q).

Poverty alleviation is a key multi-dimensional and multi-layered strategy to remove economic incentives for urban relocation. It can be achieved by (i) generating alternative income sources for rural farmers or nomadic herders, (ii) improving small-scale, agroecological agriculture and cattle-ranching methods, and (iii) enhancing vocational training in school education. To promote rural relocation, these strategies should go hand in hand with moving government institutions and infrastructure to rural areas, more equitable resource distribution of land, water, seeds, fertilizers, etc., and improving remuneration and fair prices for raw materials and agricultural products, as well as with microcredit schemes and financing projects, for instance, to provide reliable sources of clean water.

#### 3.7.2. Improving Family Physician Training and Primary Healthcare Working Conditions

Participants called for strengthening FP training as a prerequisite for building PHC capacity. FPs, as generalists, provide a wide range of healthcare services, support the development of quality PHC services, understand the environment in which communities live, and are culturally competent in responding to community concerns. Research capacity in FP training is needed, such as working with public health experts on disease surveillance in populations, investigating underlying causes of changing disease patterns in relation to climate change, and developing pathways to strengthen health systems. Systematic data collection and analysis of the nexus and disseminating analysis results could enhance FPs’ capacity to lead and advocate.

Participants had not always systematically made the connections in the nexus and recognized a need to educate themselves (quote R). This could start with participating in intersectoral forums on health implications for individuals and communities and with brainstorming about FPs’ responsibilities in response to those implications. FP curricula (and PHC in general) should include climate change and migration-related health risks in courses on determinants of health.

Health workforce plans developed by policy makers should address these curricula adaptations, improve FPs’ living and working conditions, and set minimum standards for health infrastructure. PHC workers should receive similar remuneration to other medical specialists, and other incentives such as family accommodation, school fees, and preferential selection for postgraduate programs to encourage them to continue working in rural areas (quote S).

#### 3.7.3. Health Advocacy in Primary Healthcare

Participants perceived that PHC providers could use media to disseminate information on the nexus, to lead by example and educate the people, and to raise awareness by referring to the burden for future generations when addressing climate change. FPs could sensitize politicians, policymakers, civil society, and communities about their responsibilities to protect the environment (quote T). Working as generalists, FPs may be more exposed to the negative effects of climate change and migration and may therefore be trusted voices that can raise awareness and call for action (quote U). Similarly, the wide community health worker (CHW) networks in SSA can cascade prevention strategies for newly emerging diseases resulting from climate change and migration to communities.

Participants suggested to reduce pressure on natural resources by changing cultural beliefs and practices that push women to have many children, through education and stronger family planning programs alongside better maternal and child healthcare. Instead of disease-specific vertical programs, there is a need for proactive, preventive, and curative services provided by FPs and other frontline workers such as CHWs (quote V).

#### 3.7.4. Collaboration between Health Sector and Civil Society

Collaboration between the health sector and NGOs could enhance health responses as local NGOs are in some cases better prepared for and can respond more quickly to health problems (quote W), particularly disaster relief. Such collaboration can also lead to community-level interventions to deal with nexus-related problems. Examples include waste recycling or tree planting to combat desertification (quote X).

#### 3.7.5. More Responsibilities for High-Income Countries in Supporting Intersectoral Capacity Building

Finally, some described SSA as a victim of unbalanced globalization through unfair price regulations and trade agreements with high-income countries. Examples include international timber markets linked with deforestation (quote Y) and health worker intercontinental brain drain resulting from economic migration to high-income countries, constraining SSA health system resilience (quote Z). It was stated that high-income countries should compensate affected countries, support their development, and be held more accountable, especially in how development aid is used to benefit their populations’ livelihoods.

## 4. Discussion

### 4.1. Key Findings

This study revealed rich and diversified perceptions of 30 participants on interactions in the climate change, migration, and health(care) nexus, originated from grassroots experiences in PHC in SSA. These perceived interactions were categorized as (1) migration and climate change, (2) migration related to seeking better health and healthcare, (3) health impacts related to climate change and the role of healthcare, and (4) health impacts related to migration and the role of healthcare. These relationships are complex, interacting and reinforcing one another. We visualized them as continuous feedback loops between the nexus’s dimensions, the socio-economic, institutional, and demographic context, and relevant health determinants. Participants also reflected on intersectoral capacity-building opportunities on micro, meso, macro, and supra levels: (1) developing multi-dimensional and multi-layered governance structures, (2) improving FP training and PHC working conditions, (3) health advocacy in primary healthcare, (4) collaboration between the health sector and civil society, and (5) more responsibilities for high-income countries in supporting intersectoral capacity building such as reducing intercontinental brain drain.

### 4.2. Interpretation of the Results

To our knowledge, this is the first qualitative study exploring perceptions of PHC professionals on the climate, migration, and health(care) nexus in SSA. Participants of this study viewed migration patterns in SSA as a complex phenomenon, predominantly economic and health motivated or conflict related, rather than uniquely triggered by climate change. They recognized that drivers for migration included competition for resources and the search for a dignified life, work opportunities, income, and better healthcare. Climate change impacts such as drought and floods may further catalyze these factors. These results align with previous literature which states that migration depends on local determinants of health as well as the socio-economic, political, and demographic context [1,2,3,8]. McMichael and colleagues suggest that nexus analysis should focus on migration-related health outcomes rather than on climatic events [3]. Participants consistently perceived continuous feedback loops in the nexus, mentioned its complexity and unpredictability, and acknowledged that multiple factors can simultaneously impact populations in multiple ways. Future research and policy development should recognize this complexity and heterogeneity by applying methods specifically designed for analyzing and disentangling complexity [21,22].

While (climate-related) migration may be a health-seeking strategy, participants recognized that it may also result in more health risks, which was also identified by earlier research [2,8]. Economic hardship following urban relocation prompts overcrowding, sanitary problems, poverty, drug use, and prostitution. Host health services struggle to support incoming migrants and are often not well integrated with (internationally supported) refugee health programs [23]. Additionally, health worker brain drain, driven by poor remuneration and inadequate living and working conditions, especially in rural areas, reduces the remaining workforce’s capacity and morale [12,24]. Eventually, this deteriorates health service delivery effectiveness for the entire population [24]. Therefore, climate-resilient and migration-inclusive health systems should strengthen overall PHC service delivery, and not focus solely on support for migrants affected by climate change [16]. This has been emphasized in the Astana declaration on PHC and included in the Global Sustainable Development Goals and universal health coverage [25]. Global-level policy and (low-income) country-level health plans should adopt the indicators of the PHC performance initiative framework [26] to better connect PHC with climate and migration health risks [19].

Participants recommended several intersectoral capacity-building opportunities that have been highlighted in other frameworks and publications. The WHO operational framework for climate-resilient health systems includes an integrated development agenda between local and national governments and international bodies, including multi-layered governance structures with clear strategies and co-responsibilities for health, education, social services, agriculture, food security, and poverty-alleviation sectors [16]. Poverty-alleviation strategies to remove urban migration incentives and improve rural relocation of health workers could be further explored within rural development programs [27,28]. Disaster management principles could be integrated with high-performing PHC for resilient health systems for the entire population [29].

Participants suggested ways that FP training could be expanded to incorporate aspects of the nexus in curricula, for example, the social and environmental determinants of health, or emergency responses to disasters. Authors have identified opportunities for training and research for FPs and primary care providers to strengthen the resilience of communities and to influence policy and research priorities [18,30]. Such public health and emergency medicine functions are still scarce resources in most SSA health districts [19]. FPs as clinician scientists can also document climate change and migration impacts on health (services) [18]. FP and PHC networks such as Primafamed and the African Forum for PHC (AfroPHC.org, accessed on 10 June 2021) could help conceptualize a research agenda and enable research capacity building [31]. Frontline FPs supported by established family medicine or public health researchers can address research problems at the clinical practice level and in communities such as the determinants of changes in health service utilization, the district municipality workforce, and disease outbreaks, and produce evidence on health implications of migration.

Finally, the voice of the PHC sector is a potentially impactful and yet neglected part of the environmental discourse [19]. As PHC providers such as CHWs, midwives, nurses, and FPs are highly trusted by local communities, they can sensitize national governments, private sector actors, and civil society on their responsibilities and raise public awareness on environmental issues [18]. This aligns with Schwerdtle et al. (2020), who suggest adding communications and advocacy as additional elements in the WHO operational framework [4]. PHC teams, in collaboration with NGOs, can support communities in moments of despair and coordinate climate change adaptation activities such as re-forestation, provision of clean sources of water, food security, and shelter [32,33]. Moreover, as PHC teams in different SSA countries are usually composed of people with highly diverse disciplinary backgrounds [17], other PHC disciplines such as nurses, midwives, and pharmacists may be equally well placed to contribute to the abovementioned FP responsibilities.

### 4.3. Limitations

This study has several limitations. First, recruitment of attendees at the Family Medicine Conference may mean that the study group was not representative of primary care providers or family physicians across SSA. While most participants were male, which reflects the reality of the FP population in SSA, female-specific health risks may have been underrepresented in our data. The study group also had stronger participation from East Africa, as the conference was held in Uganda. Still, our sample included participants from twelve SSA countries which allowed for a diverse representation of the different SSA regions. Second, there may have been some selection bias in that participants self-selected to join the FGDs and may have had more knowledge of or interest in the impact of climate change on migration and health(care). Yet, the aim of this qualitative research was to achieve depth of understanding (information rich) rather than have a sample that was representative of the attendees at the conferences who were working in PHC [34]. Finally, the study was designed by European researchers which may have biased the interpretation towards European views on the nexus. The research team was, nonetheless, quite diverse with scholars from multiple research disciplines, and African researchers were involved in the development of the idea, data analysis, and writing of the article.

## 5. Conclusions

This exploratory study presented a unique and novel perspective on the climate change, migration, and health(care) nexus in SSA, using perceptions from frontline FPs working in SSA. Their ideas on intersectoral capacity-building opportunities can contribute to interdisciplinary research agendas and (PHC) policy responses on national, regional, and global levels, emphasizing the urgency for and the responsibility of FPs and PHC providers to be actively developing and involved in climate-resilient and migration-inclusive health systems.

## Figures and Tables

**Table 1:** Participants’ demographic variables.

Participant	Country	Age	Gender M/F/X	Function	Rural/Urban
P1	Kenya	61	M	Family physician and academic	Rural
P2	DR Congo	54	M	Family physician and academic	Urban
P3	Nigeria	57	M	Family physician and academic	Urban
P4	Zimbabwe	49	M	Family physician, private	Urban
P5	DR Congo	56	M	Family physician	Rural/urban
P6	Uganda	61	M	Family physician, private	Urban
P7	DR Congo	44	M	Family physician	Semi-rural
P8	Nigeria	55	M	Family physician and academic	Unknown
P9	Uganda	51	F	Family physician, private	Unknown
P10	South Africa	57	M	Family physician and academic	Urban
P11	Sudan	50	M	Family Physician and academic	Unknown
P12	Nigeria	65	M	Family physician, private	Rural
P13	Nigeria	59	M	Family physician and academic	Unknown
P14	DR Congo	38	F	Family medicine trainee	Unknown
P15	DR Congo	34	M	Family physician	Unknown
P16	Kenya	38	F	Family physician	Semi-urban
P17	Rwanda	40	M	Family physician and academic	Urban
P18	Kenya	34	M	Family medicine trainee	Rural
P19	Kenya	39	M	Family physician	Unknown
P20	Uganda	42	F	Non-medical (church organization)	Unknown
P21	Zambia	54	F	Family medicine trainee	Unknown
P22	Botswana	38	F	Family medicine and academic	Rural
P23	Malawi	44	F	Family Physician and academic	Semi-urban
P24	South Africa	55	M	Family Physician and academic	Urban
P25	Uganda	70	M	Family medicine and academic	Rural/urban
P26	Uganda	19	F	Undergraduate medical student	Unknown
P27	Nigeria	59	M	Family physician and academic	Unknown
P28	Nigeria	60	F	Academic (vocational education)	Unknown
P29	Somaliland	30	M	Family medicine trainee	Rural
P30	Zimbabwe	60+	F	Family physician, private	Urban

**Table 2:** Corresponding quotes.

**Participant Quote** *Note: Words in square brackets were inserted to modify and explain the participant’s original quote.*	
** Perceptions of migration related to climate change**	
A	“We tend to have short period of rains, short but they can also be very extreme sometimes, destroying the crops. The dry season tend also to be longer and very dry, and this has actually an impact on immigration, internal immigration, agriculture is no longer interesting for many people in rural areas, we are seeing many moving now to urban settings searching for other ways to survive.” (P17, Rwanda)
B	“… for centuries we have had people whose means of livelihood is moving around with cows, they are herdsmen, they were restricted to certain parts of the country but because of desertification, they are moving further downwards towards the southern part of the country… creating clashes between them and people whose normal way of life is farming… the herdsmen are coming with their cows, destroying farms… problems of physical violence is very, very high.” (P13, Nigeria) “Climate change is putting pressure on our country for migrate, people moving down south to get pasture and all that. That is added to pressure; because … there is pressure on competition for resources between the people moving south and the people whose life is there. That is disrupting the lives of those people [whose life is there], especially in their agrarian area, it is disrupting, they cannot farm, … because somebody is going to attack them and kill them. This has also created another internally displaced people crisis. People [who lived on the farm] run away from their farms [because of the migrants] and they are moving to the cities [as IDPs].” (P8, Nigeria)
C	Several families, that I personally know have settled in the … east coast of Mozambique in Beira and just recently, with the cyclones the entire city of Beira was destroyed. Everything they had was lost. And in many of these places they [the families] came in, they brought in lots of resources, and built up a local economy and they do not function with insurance …, many times this kind of informal economy in these places are not working with that kind of support systems. So, when things happen, it destroys them, and in this case, they simply gave up and moved, and that is a nod to that economy. Going to what they thought was safer places, um, I mean, we had floods … along the coast in Durban … and these go inland where just simply rain comes and rivers flood and we do not know that kind of … water management systems, and rivers flood quite easily. … The impact on people, besides losing all their possessions, is that they do not have access to basic income, and they lost things …, so the stress of that brought in all ways … (FG2, P24, South Africa)
D	“You would see trees, green, but today scarcely no trees and the rice has made life more difficult [in the Eastern Uganda, there is rice growing but that has drained the environment completely of any green, many streams have been left muddy, so during the rainy season there is water flowing, the problem is that when the water flows and it continues to rain, nothing sinks in, in the dry season the soil just dries up]. So, people, now when they are not producing much, then they move away from that one place… they sell the rice which they have grown, because the environment has been degraded a lot, get some money and move to town.” (P25, Uganda) “I just wanted to bring up a different dimension of deforestation. We have all talked about charcoal, which is common in most of our countries. But also, the issue of cutting trees for timber and mostly this timber is being sold to Europe or to China and in my country for example there is a particular type of timber which is um ‘’Mukula’’ tree and they say that to grow that tree to a full, you know, um, a big tree, like a good size, it takes like 6 to 7 years. But those are the trees that we are busy cutting and big lots of them are being shipped out of the country. And so, um, I think as a solution to it, I would say that I mean these are the issues we should be discussing at international level, to try and ban basically, you know, or regulate the sale of this timber because the market is out there. So, if we can tackle that, at that end, maybe then the cutting basically will be reduced.” (P21, Zambia)
** Perceptions of migration related to seeking better health and/or healthcare**	
E	E.1.: “Then there is that link between migration and health and I will share a story from one of our collaborators, we collaborate with partners in health which is international NGO that yeah. Partners in health started in Rwanda to help to transform the health system in Rwanda since 2006 I think 2005–2006 and one of their strategies was to target rural areas where those were really poverty, the HIV prevalence was very high, and that is what they did, they went in those regions, particularly in the region called Kayonza in Eastern Rwanda, it is a very dry region, poverty was really there, comparing to other regions in Rwanda and the HIV prevalence was very high in that region and they targeted that region, they start providing what they called holistic care, they will not only treat patients with HIV but they will also provide food, shelter, school fees for kids where parents living with HIV. And what happened suddenly with that, they start seeing people moving from other regions to that region Kayonza coming, some of them came because they are HIV positive. Some others came because of another idea in mind, okay there they are giving food to people, they are giving houses to people, they even giving school fees to people, that is the place we should go. And then they would go there and then they will ask what the conditions are to be enrolled in that nice new program and ph-workers said okay you need to have, okay, to be HIV positive. Then, when they test them, they are not HIV positive, suddenly they cannot benefit from that, they cannot get food, shelter, housing and all the other benefits HIV positive patients were getting. What happened is that some people would just actively search for ways for to be infected. Those are anecdotal stories, but they happened. So, they could move in the peer setting being treated and get the extra benefit because for them the most important was if I can get food, house, school fees for my kid, then that would be okay, if I have HIV it’s not a problem, they will take care also of HIV.” E.2: “Starting with our own family, our mum is asthmatic, so we had to move from our rural home to an urban area where she could access health care… many families are also doing the same… even the distribution of the population in the different towns… [is] concentrated around hospitals.” (P18, Kenya)
F	“Sometimes it may not be entirely true that population would move because they are seeking healthcare, because we are assuming that the only healthcare that is there is conventional. But in Africa it is well known that traditional healthcare is right there. And people have been using it for a long time and a lot of people in very remote areas, still use other alternative medicine. And therefore, I think in our country it is not a major factor that people would migrate because they are seeking healthcare.” (P19, Kenya)
** Perceptions of health impacts related to climate change and the role of healthcare**	
G	“I mean this is epidemic, but you find that even within that same country let us say Nigeria, there are areas that have less incidences of malaria, but with this flooding that is happening in places you know that they did not used to happen, but they are now happening more and more cases coming more frequently, you know, and you are having more resistant cases, you know. So its impacting on the practice, I mean you have a practice, you know that … usually this period you spend so some number of maybe malaria or some other infectious diseases, but now you are surprised that you are having more of them, and they are even coming with more complications, so we know that that is as a result of climate change.” (P27, Nigeria) “There was a flash flood… lost a whole lot of homes … suddenly saw all sorts of things happening to the local clinic where there is a bundle of people coming in … it’s a traumatic event both physically and mentally and it had all sorts of impact on that local clinic.” (P24, South Africa) “There is also drying up of the rivers that somebody had mentioned, and already when a place where there is not much water to start off with. So recently in the outpatient setting where I work, we have seen a lot of adults with diarrhoea basically, and we are thinking, we are not quite sure, we are thinking it’s because of now water security is a problem. And they are just drinking water that is available, so just in terms of that, I think it is causing a bit of migration, people moving around trying to find where there is water.” (P22, Botswana)
H	“This year it was delayed rain, last year it was excess, so we had floods. In our setting, there is poor water sanitation, so the pit latrines get flooded, water from pit latrines mixes with the shallow wells. Most people in the rural areas would access that water, which exposes them to diseases including cholera.” (FG2, P21, Zambia)
I	“… the cyclone came … with vengeance … the roads and people’s houses were swept [away] overnight, some communities vanished completely and … bodies could not be retrieved … followed with a lot of anxiety, depression amongst relatives … in a country with a very weak health system.” (P30, Zimbabwe)
J	“I was working in a very remote area and we had to travel an awfully long distance where even vehicles could not access… we had to use motor bikes… there [were] small rivers, so water was not a problem. We had solar panels … with time the forest was cleared because of charcoal burning, the rivers dried so people moved from those towns and even nurses and doctors, including myself, we had to move to … semi-urban areas. And even some health centres were to be closed because of the health care providers not wanting [to] settle in the area.” (P18, Kenya) “When there is a landslide the need for managing the trauma is extremely high, and we are not going to get doctors being taken there, we see red cross, we try to take the ambulances. But sometimes they are not accessible at all, and then that means you need to have had people who were giving maybe nearby, already living nearby who can manage some of those cases. But most of those cases, are not, they do not have doctors, even in most of their hospitals they are not there, they may have only one doctor and may not be able to be functional. So that brings the real problem because earlier there are, I think doctors were relatively well spread out, but somebody graduated and was posted to work out in the hospital there and they went, but now it is not that easy. You know some people, some places just lack doctors, they lack nurses, and yet they were trained, challenges are of course that many people who are trained end up going out, because for greener pasture.” (P25, Uganda)
** Perceptions of health impacts related to migration and the role of healthcare**	
K	“I think one of the other things that we’ve noticed is that people are moving from, nomadic people and people taking care of their cattle, people are moving from what we call cattle-posts into villages that were previously not documented, so they are creating these new villages and then they need healthcare access, and planning for that sort of becomes difficult.” (P22 Botswana)
L	“… So I work in an urban setting but also supervise in rural settings, we see a lot of childhood asthma and much more than previous years and I think, I don’t know who said that, they move to the city indeed because that’s where there is better health care, … and I think there is more pollution in Nairobi than there is in the rural areas and I am not sure if that would be very beneficial to their asthma … ” (P16, Kenya)
M	“I think the situation in South Africa is obviously internal migration from more rural areas to urban areas. I think that it is fuelled mainly by economic … I think people are looking for better economic possibilities rather than related to climate change. And then I think there are refugees who come to the country. So, there was a big economic meltdown in Zimbabwe and so many Zimbabweans came to south Africa. I think that people come to south Africa from other conflict areas, so the DRC, Eritrea, Sudan those kinds of things. Now one of the issues that is caused in South Africa is xenophobia and so there is black-on-black violence where the perception is that brothers and sisters from other parts of Africa are stealing jobs from South Africans and so that has created a huge issue and lots of people have in fact moved back, so I know a lot of people moved back to Malawi for instance after a wave of xenophobic attacks within the country.” (P10, South Africa)
N	“In Nigeria too we are noticing, apart from the known physical conditions, people, there is increasing in suicides, people are jumping over into the bridges in the bigger cities like Lagos and trying to. Someone had tempted to suicide are also inside. I also noticed increased drug use of psychotic drugs among people because of the pressure, they feel inadequate to meet of the standards, because sometimes, you have the whole family, have somebody who is well off compared to others and must cater for all these other rural relatives at his place who got displaced, lost their jobs, or ran away from their jobs and all those. And then the pressure, he cannot just cope up, and just decide to take his life and go into depression and many other start alcohols and all those things are increasing.” (P8, Nigeria)
O	“But that is not really put into practice, so we have this seasonal overwork of health personnel. But there is also another one, this one is migration, this migration of health personnel out of the country, right? Because of many other pulling factors and that, the impact of that migration, is that there is shortage of health care personnel and we have more work to do and even the health personnel themselves get, they get ill more often and the health system is really overstretched … And unless we are able to produce more health personnel or attract them back, then we are going to have a big crisis in the very near future in the health system.” (FG3, P27, Nigeria)
** Complex, interacting, and continuous feedback loops**	
P	“We have had cyclones before, but this one was horrific. And what pains me mostly is the government did nothing about warning those people to move to higher safer grounds. When the cyclone came, it came really with vengeance and it struck down the roads and people’s houses were swept over, overnight, some communities vanished completely and some of the bodies could not be retrieved; they floated all the way to Mozambique and things like that. So that followed with a lot of anxiety, depression amongst relatives and unfortunately that happened in a country with a very weak health system right now.” “To try and cope and help those people was not easy. We got a high prevalence of HIV in Zimbabwe, so some of those family members whose homesteads were swept away, and they survived, the following day they did not have access to their ARVS-medication, so you can imagine what that is going to cause in the end. The government took too long to respond to the catastrophe. So, people were not having clean water, which means we are going to have a whole lot of water borne diseases coming from that place. My main worry right now is it is going to take a long time before the infrastructure is repaired and the clinics are revived in that area. What if another catastrophe hits the same area? You can imagine people migrating from that region to the nearer one, which is already having a weak health system. How is that health system going to cope? There is going to be a whole lot of infectious diseases that are going to arise around from that. And we have lost a lot of doctors, also they leave the country to look for greener pastures.” (FG30, P30, Zimbabwe)
** Perceived opportunities and conditions for intersectoral capacity building**	
Q	“At a country level, for me, we need an integrated development agenda, where all those issues can, people can think together about all those issues.” (P17, Rwanda) “Say for instance as a family physician, I probably can get invited into the county health committee where we sit down and talk about our health issues in the county and prioritize and say you know we need to put resources in this and this. That’s a forum where I sit not only with just doctors, but I sit with administrators, you know the person in charge of forest there and the one in charge of sanitation is there, the district, the county commissioner sits there and these are areas where we can have you know like a plan of action, and say look here the two communities down there fighting, partly because they don’t have water, but the forest that we have near the Embuguti has got people who have moved in and they are cutting down the forest, so is it possible maybe from the administration side, for you to reign in these people or even offer opportunities for us to sit with these people and try to explain to them that their actions are probably contributing to their problems.” (P19, Kenya).
R	“…Educated as I am… it takes a lot for me to connect climatic change with health … and migration… we don’t attribute what is happening now to what we have done.” (P23, Malawi)
S	“The money may not be there, there could be some other form of incentive, you could pay both of them the same amount and then say maybe to the one who goes to the rural area your child will be entitled maybe to free education, you know, that’s one way to look at it, or you say you know to the doctors who went to the rural area that if you have been there for two years you are the candidate to go into post-graduation training and the government maybe will pay for you and that is an incentive to keep people down there, it doesn’t have to be cash.” (P28, Nigeria)
T	“But I think on our side we need to educate people that when you cut a tree you probably should think of your great great great grandchildren. Maybe cut one and plant one or plant two.” (P19, Kenya) “Education is something that can be done by anybody even as a family physician yeah, I think we have talked about many things that you know climate change is impacting in terms of causing health problems and even as family physician one of our roles is we are educators. And we might be able to do it in individual capacities but given a forum where we might be able to influence change, then we can show the causality or the association between you know climatic change and some of the disease pattern that we are seeing and these are opportunities for capacity building, they’re opportunities for educating.” (P19, Kenya)
U	“…we can as family doctors mobilize other sectors within our communities and get together and own … our problems and try to work towards … overcom(ing) the adversity.” (P1, Kenya) “I quickly want to touch on the advocacy people have talked about. And being a family physician, I think we, one of our roles is to be an advocate and deliberate and an active one. And I think being at the central role of being a family physician where you’re in contact with, you know, everyone, most stakeholders that are in health. And that’s an opportunity to make a statement. An example what happens in Malawi, is the society of medical doctors starting from the other year, every year they set a tree planting day and make big media, you know visibility about it and make a statement about tree planting.” (P23, Malawi).
V	“The problem is that at the moment it’s very verticalized programs… we need to have a team-based effort where there is relatively strong skill of a family doctor who is working with the defined population, where they, together with others shifting big parts of the team, including community workers, can have a much more pro-active approach to the community.” (P22, Botswana)
W	“In capacity building I think we need to think about the NGO’s, the non-governmental organizations; because usually it occurs in crisis where the health system is not able to absorb the shock. So, we need to build capacity building for the organizations, for the local organizations and for the western organizations also; but usually the local organizations are more prepared and can catch more quickly for the problems. More for the, uh, we need a community participation or capacity building inside, the normal people of the community, how to deal with the impact of climate change inside the community and to lead the community towards the changes which are happening and how to deal with it.” (P11, Sudan).
X	“One of our roles is to be an advocate, a deliberate and active one… in contact with … most stakeholders that are in health. An example what happens in Malawi, is the society of medical doctors starting from the other year, every year they set a tree planting day and make big media, you know visibility about it and make a statement about tree planting.” (P23, Malawi)
Y	“I just wanted to bring up a different dimension of deforestation. We have all talked about charcoal, which is common in most of our countries. But also, the issue of cutting trees for timber and mostly this timber is being sold to Europe or to China and in my country for example there is a particular type of timber which is um ‘Mukula’ tree and they say that to grow that tree to a full, you know, um, a big tree, like a good size, it takes like 6 to 7 years. But those are the trees that we are busy cutting and big lots of them are being shipped out of the country. And so, um, I think as a solution to it, I would say that I mean these are the issues we should be discussing at international level, to try and ban basically, you know, or regulate the sale of this timber because the market is out there. So, if we can tackle that, at that end, maybe then the cutting basically will be reduced.” (P21, Zambia).
Z	“But still, people have been, if you go back to political economics. People have really drained Africa. Beginning with the trades, slave trade and all that. If Europe can sincerely, you know, today they do that by brain drain, where they encourage young ones to come over and then real messing here where they go back there to practice. … If Europe really knew it they would stop those drainage. We have more than enough to be happy and to build this country, and build Africa. In fact, I’m encouraged when I hear what Africans are doing in America and France, I mean they are the best stories, and these stories are told all over the whole place and still they cannot do it in their own place because this place is so depleted (P12, Nigeria).

## Data Availability

The data that support the findings of this study are available from the corresponding author upon reasonable request.

## References

[1] Schütte S., Gemenne F., Zaman M., Flahault A., Depoux A. (2018). Connecting planetary health, climate change, and migration. Lancet Planet. Health.

[2] Mcmichael C., Barnett J., Mcmichael A.J. (2012). Review an ill wind? Climate change, migration, and health. Environ. Health Perspect..

[3] McMichael C. (2020). Human mobility, climate change, and health: Unpacking the connections. Lancet Planet. Health.

[4] Schwerdtle P.N., Stockemer J., Bowen K.J., Sauerborn R., McMichael C., Danquah I. (2020). A meta-synthesis of policy recommendations regarding human mobility in the context of climate change. Int. J. Environ. Res. Public Health.

[5] Kula N., Haines A., Fryatt R. (2013). Reducing vulnerability to climate change in Sub-Saharan Africa: The need for better evidence. PLoS Med..

[6] Falco C., Donzelli F., Olper A. (2018). Climate change, agriculture and migration: A survey. Sustainability.

[7] Mastrorillo M., Licker R., Bohra-Mishra P., Fagiolo G., Estes L.D., Oppenheimer M. (2016). The influence of climate variability on internal migration flows in South Africa. Glob. Environ. Chang..

[8] Watts N., Amann M., Arnell N., Ayeb-Karlsson S., Belesova K., Boykoff M., Byass P., Cai W., Campbell-Lendrum D., Capstick S. (2019). The 2019 report of The Lancet Countdown on health and climate change: Ensuring that the health of a child born today is not defined by a changing climate. Lancet.

[9] Schwerdtle P., Bowen K., McMichael C. (2018). The health impacts of climate-related migration. BMC Med..

[10] Watts N., Amann M., Arnell N., Ayeb-Karlsson S., Belesova K., Berry H., Bouley T., Boykoff M., Byass P., Cai W. (2018). The 2018 report of the Lancet Countdown on health and climate change: Shaping the health of nations for centuries to come. Lancet.

[11] Abubakar I., Aldridge R.W., Devakumar D., Orcutt M., Burns R., Barreto M.L., Dhavan P., Fouad F.M., Groce N., Guo Y. (2018). The UCL–Lancet Commission on Migration and Health: The health of a world on the move. Lancet.

[12] Agyepong I.A., Sewankambo N., Binagwaho A., Coll-Seck A.M., Corrah T., Ezeh A., Fekadu A., Kilonzo N., Lamptey P., Masiye F. (2017). The path to longer and healthier lives for all Africans by 2030: The Lancet Commission on the future of health in sub-Saharan Africa. Lancet.

[13] Marten M.G., Sullivan N. (2020). Hospital side hustles: Funding conundrums and perverse incentives in Tanzania’s publicly-funded health sector. Soc. Sci. Med..

[14] Pfeiffer J., Gimbel S., Chilundo B., Gloyd S., Chapman R., Sherr K. (2017). Austerity and the “sector-wide approach” to health: The Mozambique experience. Soc. Sci. Med..

[15] (2019). Expert Panel on Effective Ways of Investing in Health (EXPH) Expert Panel’s Reflection on Priorities for the Future of Healthcare in the European Union. https://ec.europa.eu/health/sites/default/files/expert_panel/docs/2019_brainstorming_en.pdf.

[16] WHO (2019). Operational Framework for Building Climate Resilient Health Systems. http://www.who.int/globalchange/publications/building-climate-resilient-health-systems/en/.

[17] Mash R., Howe A., Olayemi O., Makwero M., Ray S., Zerihun M., Gyuse A., Goodyear-Smith F. (2018). Reflections on family medicine and primary healthcare in sub-Saharan Africa. BMJ Glob. Health.

[18] Xie E., de Barros E.F., Abelsohn A., Stein A.T., Haines A. (2018). Challenges and opportunities in planetary health for primary care providers. Lancet Planet. Health.

[19] Kadandale S., Marten R., Dalglish S.L., Rajan D., Hipgrave D.B. (2020). Primary health care and the climate crisis. Bull. World Heal. Organ..

[20] Chersich M.F., Wright C.Y. (2019). Climate change adaptation in South Africa: A case study on the role of the health sector. Glob. Heal..

[21] Tacoli C., Mabala R. (2010). Exploring mobility and migration in the context of rural—urban linkages: Why gender and generation matter. Environ. Urban..

[22] Holland J.H. (2006). Studying complex adaptive systems. J. Syst. Sci. Complex..

[23] Orach C.G., De Brouwere V. (2005). Integrating refugee and host health services in West Nile districts, Uganda. Health Policy Plan..

[24] WHO Guidance for Climate Resilient and Environmentally Sustainable Health Care Facilities. https://www.who.int/publications/i/item/9789240012226.

[25] Rasanathan K., Evans T.G. (2020). Primary health care, the Declaration of Astana and COVID-19. Bull. World Heal. Organ..

[26] The PHCPI Conceptual Framework. https://improvingphc.org/phcpi-conceptual-framework.

[27] Zadawa A.N., Omran A., Omran A., Schwarz-Herion O. (2020). Rural development in Africa: Challenges and opportunities. Sustaining Our Environment for Better Future.

[28] Lafleur J., Romero M. (2018). Combining transnational and intersectional approaches to immigrants’ social protection: The case of Andean families’ access to health. Comp. Migr. Stud..

[29] Jewell B.L., Mudimu E., Stover J., Brink D.T., Phillips A.N., Smith A.J., Martin-Hughes R., Teng Y., Glaubius R., Mahiane S.G. (2020). Potential effects of disruption to HIV programmes in sub-Saharan Africa caused by COVID-19: Results from multiple mathematical models. Lancet HIV.

[30] Flinkenflögel M., Sethlare V., Cubaka V.K., Makasa M., Guyse A., De Maeseneer J. (2020). A scoping review on family medicine in sub-Saharan Africa: Practice, positioning and impact in African health care systems. Hum. Resour. Health.

[31] De Maeseneer J. (2017). Twenty years of primafamed network in Africa: Looking back at the future. Afr. J. Prim. Health Care Fam. Med..

[32] Olympia R.P., Rivera R., Heverley S., Anyanwu U., Gregorits M. (2010). Natural disasters and mass-casualty events affecting children and families: A description of emergency preparedness and the role of the primary care physician. Clin. Pediatr..

[33] Hogg W., Huston P., Martin C., Soto E. (2006). Enhancing public health response to respiratory epidemics: Are family physicians ready and willing to help?. Can. Fam. Physician.

[34] Patton M.Q. (2002). Qualitative Research and Evaluation Methods.

